# Overground walking training with the *i*-Walker, a robotic servo-assistive device, enhances balance in patients with subacute stroke: a randomized controlled trial

**DOI:** 10.1186/s12984-016-0155-4

**Published:** 2016-05-26

**Authors:** Giovanni Morone, Roberta Annicchiarico, Marco Iosa, Alessia Federici, Stefano Paolucci, Ulises Cortés, Carlo Caltagirone

**Affiliations:** Clinical Laboratory of Experimental Neurorehabilitation, I.R.C.C.S, Fondazione Santa Lucia, Rome, Italy; Behavioural and Clinical Neurology Laboratory, I.R.C.C.S, Fondazione Santa Lucia, Rome, Italy; Department of Neuroscience, “Tor Vergata” University, Rome, Italy; Universitat Politècnica de Catalunya Barcelona, Barcelona, Spain

**Keywords:** *i*-Walker, Floor training, Robotic-assisted therapy, Rehabilitation, Gait

## Abstract

**Background:**

Patients affected by mild stroke benefit more from physiological overground walking training than walking-like training performed in place using specific devices. The aim of the study was to evaluate the effects of overground robotic walking training performed with the servo-assistive robotic rollator (*i*-Walker) on walking, balance, gait stability and falls in a community setting in patients with mild subacute stroke.

**Methods:**

Forty-four patients were randomly assigned to two different groups that received the same therapy in two daily 40-min sessions 5 days a week for 4 weeks. Twenty sessions of standard therapy were performed by both groups. In the other 20 sessions the subjects enrolled in the *i*-Walker-Group (iWG) performed with the *i*-Walker and the Control-Group patients (CG) performed the same amount of conventional walking oriented therapy. Clinical and instrumented gait assessments were made pre- and post-treatment. The follow-up observation consisted of recording the number of fallers in the community setting after 6 months.

**Results:**

Treatment effectiveness was higher in the iWG group in terms of balance improvement (Tinetti: 68.4 ± 27.6 % vs. 48.1 ± 33.9 %, *p* = 0.033) and 10-m and 6-min timed walking tests (significant interaction between group and time: *F*(1,40) = 14.252, *p* = 0.001; and *F*(1,40) = 7.883, *p* = 0.008, respectively). When measured, latero-lateral upper body accelerations were reduced in iWG (*F* = 4.727, *p* = 0.036), suggesting increased gait stability, which was supported by a reduced number of falls at home.

**Conclusions:**

A robotic servo-assisted *i*-Walker improved walking performance and balance in patients affected by mild/moderate stroke, leading to increased gait stability and reduced falls in the community.

**Trial registration:**

This study was registered on anzctr.org.au (July 1, 2015; ACTRN12615000681550).

## Background

Stroke is one of the leading causes of severe disability in the Western world [1]. One crucial goal in rehabilitating patients affected by stroke is to restore mobility so that patients can regain independent living and walking [2]. Up to 88 % of people affected by stroke experience hemiparesis with gait and balance disorders that persist even in the chronic phase [3, 4].

Most individuals who have suffered a stroke have asymmetric posture with resultant balance and gait dysfunction. Consequently, their performance of the activities of daily living is reduced [5] and they have an increased risk of experiencing one or more falls [6]. In particular, balance deficits in patients affected by stroke result from reduced postural control [7] and less coordinated responses to both self-induced and external balance perturbations [6]. Thus, restoring autonomous gait and recovering balance are challenges in the rehabilitation of patients with stroke [8]. Interventions aimed at overcoming gait and balance deficits should increase patients’ independence in the activities of daily living and help prevent falls.

Repetitive task-specific exercise programs have been shown to be effective in reducing balance disorders and restoring gait [9]. Technology could improve these programs and facilitate plasticity- related recovery by increasing sensory feedback and supporting the motor system [10]. Much of the current evidence supports body weight-supported technological devices, either walking overground or on a treadmill, to encourage upright postural control, normal arm swing and optimal dynamic balance [11–13]. However, contrasting results regarding robotic-assisted therapy have recently been reported concerning their potential benefits in balance and walking recovery in post-stroke patients [14]. These contrasting results might be due to the different severity of patients. Indeed, more severe stroke patients might benefit more from robotic therapy due to their difficulty in performing overground walking training [15, 16]. Conversely, less severely affected patients, who are able to walk with little assistance, might benefit more from balance and walking training on the floor with less constriction and more physiological sensory motor feedback, i.e., in a challenging condition and in a context closer to that of daily living. This hypothesis was supported by results of a large randomized trial (LEAPS Study) in patients with stroke in whom home exercises with a physiotherapist were more effective in increasing balance and reducing falls than electromechanical locomotor training [17, 18]. In neurologically impaired populations overground walking training can be performed using a walker to improve stability and increase walking capacity. This simple, beneficial and economic device may not be used appropriately to maximize function due to severity of motor deficits that are strongly asymmetric. In these patients a hemi-walker or quad cane may be needed depending on the balance impairment [19]. Further, using assistive devices in the acute and sub-acute stages of rehabilition following stroke is not supported in the literature because these compensatory strategies might limit neuroplasticity [20].

In this study, we set out to combine the promising advances in assistive technology [21] with physiological training aimed at improving mobility and balance. To accomplish this, we used a robotic device with embedded intelligence, i.e., the *i*-Walker. The objective of this device is to promote upright control and walking in people with mild/moderate stroke. Furthermore, it can be used either for training or as an assistive device. Other advantages are related to the fact that the *i-*Walker is not expensive or cumbersome and can easily be maneuvered by an individual. The *i*-Walker has almost the same dimensions as the rollator. It is, in fact, a robotic rollator (walker with 4 wheels) that integrates sensors and actuators able to provide asymmetrical assistance as needed during walking. It uses a standard rollator frame modified for this purpose. Actuators are two hub motors integrated in the rear wheels and are used for braking or helping the user. Sensors are arranged in the frame to detect forces, tilt and movement. An integrated battery supplies power. Finally, a network of distributed micro controllers drives the system and records and provides information to the therapists. The *i*-Walker passively detects the force imposed by the user on the handles through its sensors; thus, it is possible to determine and adjust the amount of help each motor should be giving to the side with a deficit [22].

The primary aim of this study was to evaluate changes in walking performance (i.e. gait velocity and gait capacity) using the *i*-Walker with respect to conventional walking-oriented therapy. The secondary aim was to study how *i*-Walker training affects balance, stability of walking and the incidence of falls in the community.

## Methods

### Participants

We considered for inclusion in this study consecutive inpatients who had recently suffered strokes and had been admitted to two different Neurorehabilitation Units of Santa Lucia Foundation IRCCS during their first three weeks of hospitalization in the period between March 2012 and December 2013. The independent Ethical Board of the Santa Lucia Foundation approved the study protocol (CE/AG4-PROG.101-135) and written informed consent was obtained from each patient and/or a relative.

Inclusion criteria were: hemiparesis caused by a first-ever unilateral stroke, subacute phase (<90 days from stroke), age between 18 and 80 years, ability to perform assisted walking training on the parallel bar (Functional Ambulation Classification [23], FAC ≥ 2), presence of some degree of muscular activity in each shoulder/elbow/hand (Medical Research Council scale [24] MRC ≥ 3). Exclusion criteria included: concomitant chronic disabling pathologies, severe spasticity (defined as score ≥4 for arm or leg on the modified Ashworth Scale [25]), moderate/severe cognitive decline (Mini-Mental State Estimation [26], MMSE score < 24) and presence of severe hemispatial neglect as evaluated by a neuropsychologist (i.e., patients needing rehabilitation for neglect were excluded).

### Intervention

The trial was designed as a prospective randomized controlled trial based on CONSORT guidelines. After randomization, which was carried out using a random computer-generated list, patients were consecutively assigned to one of the two groups. Allocation was concealed from both patients and physiotherapists; only a non-clinical experimenter who was not involved in the treatments had access to the randomized lists. All patients received two daily 40-min sessions of therapy, 5 days a week for 4 weeks, in a one-one mode. The control group (CG) performed 40 sessions of conventional walking-oriented therapy. The first daily session (i.e., 20 sessions, 40 min per session, 5 times a week for 4 weeks) consisted of overground training for ambulation exercises on the parallel bars for control and movement of the lower limb load, exercises for control of the trunk and pelvis and walking exercises of increasing difficulty on the ground. Help provided by the therapists and aids (i.e. canes, tripods or walkers) was allowed. The therapists decided which was the least restrictive assistive device and used it for gait training. The second daily therapy session was focused on exercises for hand recovery, tone control and improvement of global ability.

The i-Walker group performed one daily conventional walking training using a servo-assistive robotic walker supervised by a physiotherapist (20 sessions, 40 min per session), 5 times a week for 4 weeks. Similar to the control group, the patients’ second daily session of therapy was focused on exercises for hand recovery, tone control and improvement of global ability.

### Assessment

The evaluations were made in three steps: (T0) pre-treatment, corresponding to the time when the patient began the walking training using the parallel bars (control group) or with the *i*-Walker (experimental group); (T1) post-treatment, corresponding to the end of the 4-week walking training period; (T2) follow-up, corresponding to the 6 months after T1 during which the number of falls that occurred in the community was self-reported. All subjects had the baseline ability of being able to walk in inside the parallel bars. Once subjects had this ability, they were included in the research protocol. In accordance with the Prevention of Falls Network Europe, a fall was defined as an unexpected event in which the subject comes to rest on the ground, floor or his/her centre of mass comes to a lower level [27].

A blinded assessor evaluated training efficacy outcomes at T0 and T1. The primary outcome measure was walking capacity measured by the Six-Minute Walk Test (6MWT), [28] self-selected walking speed with the Ten Meter Walk Test (10MWT) [29]. Secondary outcomes included balance and gait assessment made using Tinetti’s Scale [30], spasticity assessed with a modified version of the Ashworth scale (sum of the six districts regarding upper and lower limbs), global ability measured with the Barthel Index (BI), [31] and global impairment assessed with the Canadian Neurological scale.

Other secondary outcomes were measured using instrumented assessment of upright gait stability.

Upright gait stability has been defined as the capacity to minimize upper body oscillations and absorb jerks, bumps, shakes and fluctuations despite broad and fast movements of the lower limbs during locomotion [32]. Hence, upright gait is stable when upper body accelerations are minimized and smoothed. Accelerations were measured while patients walked a short distance [33] inside or outside parallel bars. They were asked to stand on a line marked on the floor and walk straight for 4 m at a self-selected speed until they arrived at another line on the floor. Tests were performed inside parallel bars (with patients being able to touch the bars) or outside them, i.e. during normal overground walking under the strict supervision of the therapist (a light touch was allowed). During the test, patients wore an elastic belt that contained a wearable triaxial accelerometer placed at the level of the L2/L3 spinous process and fixed with an elastic band (FreeSense®, Sensorize s.r.l., Rome; sampling frequency = 100 Hz, weight = 93 g) to measure accelerations along the three body axes (antero-posterior, AP; latero-lateral, LL; and cranio-caudal, CC). The accelerometric signals were analysed after their mean value subtraction and after low-pass filtering at 20Hz, and their root mean square (RMS) was computed. RMS is a measure of acceleration dispersion (which coincides with the standard deviation because of signal mean subtraction) that provides information about upright gait instability. As the RMS of acceleration is strictly dependent on walking speed, we normalized the values of RMS-AP and RMS-LL with respect to those of RMS-CC by using the inverse of their percentage ratio as the indicator of stability, in accordance with previous studies of patients with stroke [33] or other pathologies [34].

### i-Walker

**Fig. 1:**
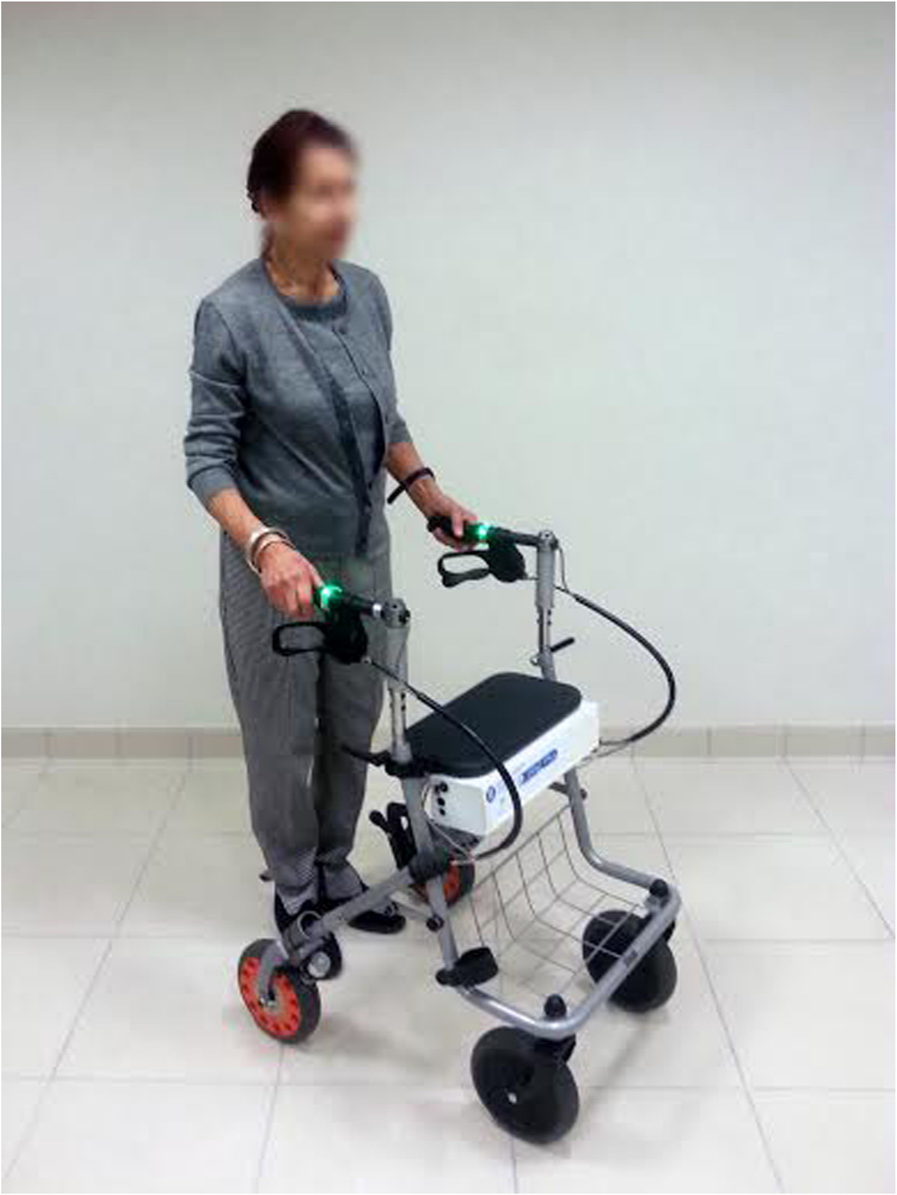
The *i*-Walker (Signed informed consent was provided by the patient for publication of this picture)

In Spain the *i*-Walker is registered as medical electrical equipment (reg. number 477/13/EC). The *i-*Walker was semi-industrial prototypes produced at Universitat Politècnica de Catalunya, with partial UE funding and purchased by the pilots. As illustrated in Fig. 1, the *i*-Walker is a robotic rollator that integrates sensors and actuators [35]. It uses a standard 4-wheeled Rollator AD100 walker frame sized 500 mm (W) × 600 mm (L) × 850 mm (H) modified for this purpose. Actuators are two hub motors, 100 mm in diameter, that are integrated in the rear wheels and are used for braking or helping the user. The device also has two modified handlebars with brake handles and force measurement, 32 strain gauges mounted in 8 bridges to measure handlebar forces and normal wheel forces, sensors arranged in the frame to detect forces, tilt and movement and an integrated battery that supplies power. The *i*-Walker detects the force imposed by the user on the handles through its sensors, so it is possible to determine and adjust the amount of help that each motor should be giving to the side with a deficit. The *i*-Walker provides no pulling force, but only assists in pushing the device forward: the *i*-Walker applies appropriate compensatory force through its motors only when pushing forces are detected through the handles. The amount of support provided by the *i*-Walker is modifiable to allow therapists to adjust support to maximize patients’ participation in walking.

The following services are provided by the *i*-Walker: (i) active motor assistance to compensate for lack of muscle force on climbs; (ii) active brake assistance to compensate for lack of muscle force on descents; (iii) active differential assistance to compensate for asymmetric muscle force; (iv) recording of sensor measurements and actuator activities for later evaluation.

In this study we focussed on point (iii), i.e. active differential assistance to compensate for asymmetric muscle force. During training the amount of assistance (i.e. braking force in each hand) was reduced by the team following this principle: (i) assistance as needed, (ii) progressive assistance reduction, (iii) safety concerns, (iv) patients’ ability to drive the device and (v) affected leg and arm increase in spasticity.

### Statistical analysis

As clinical scores are ordinal measures, they were treated with non-parametric statistics using the Wilcoxon signed ranks test for within group analyses and the Mann-Whitney *u*-test for between group analyses. The sample was selected in accordance with the criteria of previous studies that analysed the use of robotic devices in walking training [15, 36] and with a Phase 2, stage III motor intervention [37]. Furthermore, improvements with respect to the baseline were analysed in addition to the raw data. Effectiveness of the intervention was computed for clinical scale scores as the percentage of improvement made with respect to the maximum achievable improvement, i.e. (final score – initial score)/(maximum clinical scale score – initial score) * 100 [38–41]. Percentage values were treated as continuous measures and hence managed with parametric statistics.

As instrumented timed walking tests and accelerometer data were continuous measures, they were treated with parametric statistics using mixed-model repeated measure analysis of variance (RM-Anova) and Bonferroni correction was applied for post-hoc analyses. For these variables, percentage improvement was evaluated as: (final measure – initial measure)/initial measure * 100. For the 10MWT we also computed the minimal clinical importance difference (MCID) [42]. The odds ratio (OR) and relevant 95 % confidence interval (CI95 %) were computed to determine how many subjects achieved the MCID and to assess exposure to the risk of falling in the two groups of patients. The statistical significance of ORs was tested with the chi-squared test. SPSS 17.0 was used for statistical analysis. The threshold of significance was set at 0.05 for all tests.

## Results

**Fig. 2:**
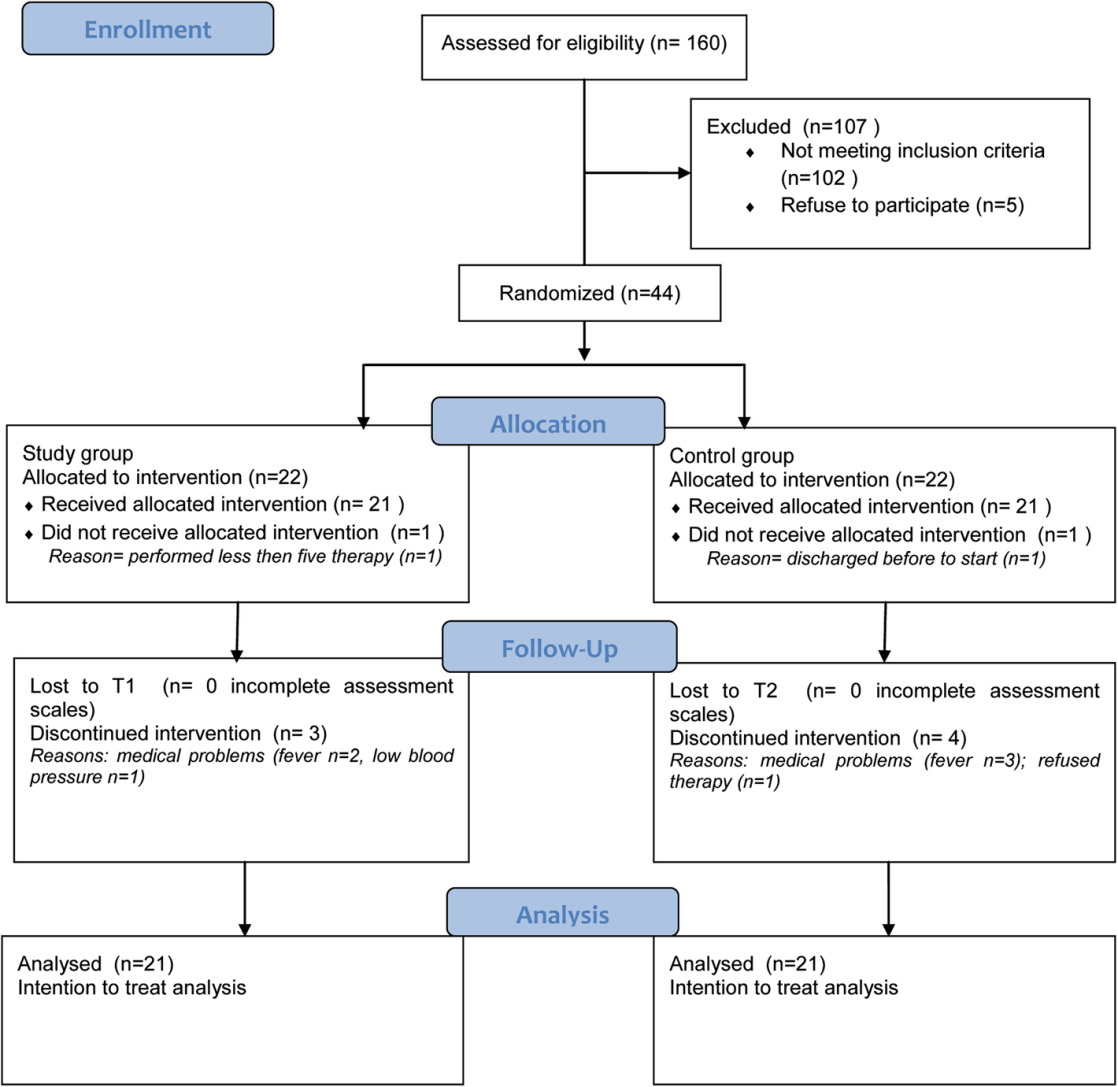
CONSORT Flow chart

Between March 2012 and December 2013, 44 out of 160 screened patients were enrolled in the study; they were randomly assigned to groups and evaluated. Two patients, one in the *i*WG and one in the CG were dropped, as shown in Fig. 2 (consort study flow chart). Demographic and clinical characteristics of the patients are presented in Table 1. The two groups were not significantly different in terms of demographic and clinical characteristics at T0, confirming the similar deficits of the two groups at baseline.

**Table 1:** Demographic and clinical characteristics of the enrolled sample at baseline (T0)

Characteristics	*i*-Walker group (*n* = 21)	Control group (*n* = 21)	Comparison (*p*-value)
Age [years]	61.50 ± 10.97	64.09 ± 16.27	0.326
Side of paresis dx/sx	12/9	13/8	0.753
Gender m/f	16/5	12/9	0.190
Ischaemic/Haemorrhagic	18/3	14/7	0.147
MMSE	26.50 ± 3.32	25.68 ± 5.61	0.576
Time onset	69.20 ± 28.93	59.68 ± 36.03	0.147
Tinetti	15.00 ± 4.54	17.09 ± 6.62	0.221
FAC	2.10 ± 0.31	2.14 ± 0.49	0.720
CNS	8.10 ± 0.97	8.52 ± 1.84	0.130
Barthel Index	64.10 ± 19.17	67.82 ± 19.83	0.632
GDS	6.50 ± 3.98	6.27 ± 4.24	0.742

Mann-Whitney *u*-test was used to compare i-Walker Group and Control Group scale scores and *χ* ^2^-test to compare side, gender and type of stroke distributions

*MMSE* mini-mental state examination, *FAC* functional ambulation category, *CNS* Canadian neurological scale, *GDS* geriatric depression scale

### Walking performance

An analysis of variance on performances at 10MWT showed that both groups improved, but the iWG showed a greater main within subject effect of time than the CG: *F*(1,40) = 37.763, *p* < 0.001; significant interaction between group and time: *F*(1,40) = 14.252, *p* = 0.001; main effect of group: *F*(1,40) = 0.451, *p* = 0.506). In fact, the percentage reduction of the time spent to complete the 10mWT was 44.8 ± 16.3 % in *i*-WG and 17.7 ± 13.2 % in CG (*p* < 0.001).

**Fig. 3:**
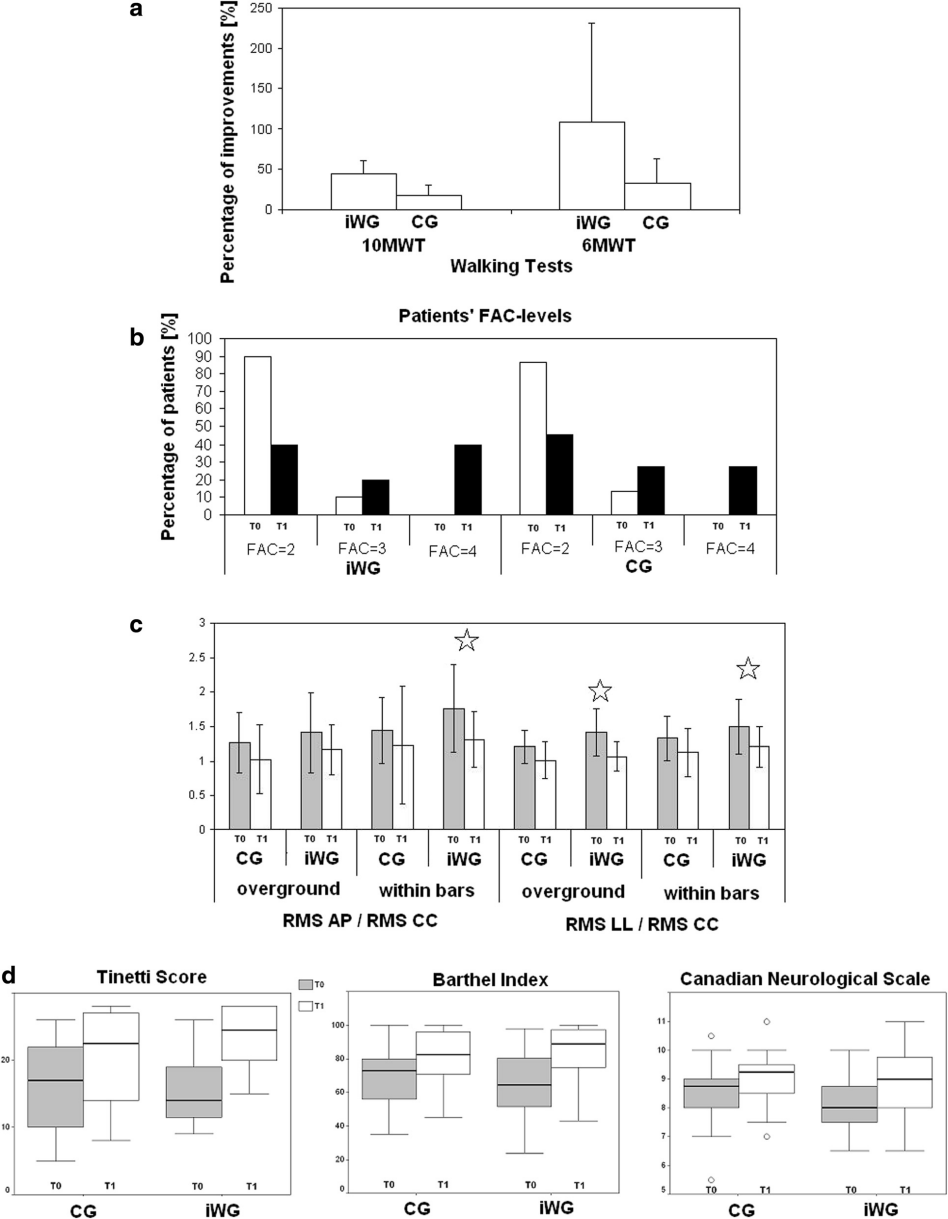
**a** Mean and standard deviation for the percentage improvements (walking time reduction for 10-m walking test, 10MWT and walking distance improvement for 6-min walking test, 6MWT) in *i*-Walker group (iWG, dotted columns) and control group (CG, grey columns). **b** Box-plot of clinical scores for control group (CG, in the left of plots) and iWalker group (iWG, in the right of plots), pre (T0, grey boxes) and post (T1, white boxes) rehabilitation. The boxes show the lower quartile, median (bold line) and upper quartile values, the whiskers represent the most extreme values within 1.5 times the interquartile range from the ends of the box, the circles represent the outliers (data with values beyond the ends of the whiskers). **c** The percentage of patients divided by FAC-level at T0 (white bars) and T1 (black bars) for iWG (on the left) and CG (on the right). **d** Upright gait stability. Normalized adimensional values of anteroposterior and laterolateral acceleration RMS pre-rehabilitation (T0, grey bars) and post-rehabilitation (T1, white bars) for control group (CG) and *i-*Walker group (iWG). Stars indicate a statistically significant difference (*p* < 0.006)

Analogously, also the distance walked during the 6MWT significantly improved in both groups, but more in the *i*-WG (main effect: *F*(1,40) = 72.087, *p* < 0.001; interaction: *F*(1,40) = 7.883, *p* = 0.008; main group effect: *F*(1,40) = 0.360, *p* = 0.552). In fact, the percentage improvement was 109.2 ± 121.6 % in the *i*-WG and 32.6 ± 30.5 % in the CG (*p* = 0.007) (see Fig. 3).

The number of patients who showed an increase from the baseline in the 10MWT distance and who reached a minimal clinically important difference (MCID), defined as ≥0.16 m/s. was 13 in the *i-*WG and 5 in the CG (OR = 6.31, IC95 % = 1.63–24.50, *p* = 0.0057).

### Clinical assessment

In both groups all clinical scale scores significantly improved from T0 to T1 (*p* < 0.05) with the exception of the Ashworth score at ankle level in the CG (*p* = 0.157) and the Ashworth score at hip level, which did not change in either the *i*-WG (*p* = 0.180) or the CG (*p* = 0.317). Due to the high variability among subjects, no differences were found at T1 in terms of raw clinical scores. However, when these scores were normalized using effectiveness for reducing inter-individual variability, many significant changes emerged. The parameter effectiveness of the Tinetti-score was significantly higher in the *i*-WG (68.4 ± 27.6 %) with respect to the CG (48.1 ± 33.9 %, *p* = 0.033).

Effectiveness computed on the other clinical scores was close to the statistically significant threshold for the BI score (*i*-WG vs. CG: *p* = 0.076) but far from this threshold for the other scores (see Fig. 3).

### Upright trunk stability measured by accelerometers

The RM-Anova performed on the data of the normalized trunk accelerations showed significant main effects of condition and time (i.e., walking inside or outside parallel bars, *F* = 11.483, *p* = 0.002; pre-post rehabilitation, *F* = 26.542, *p* < 0.001). The main effect of group was not statistically significant (*F* = 2.438, *p* = 0.127). The most interesting result was the significant effect of the interaction among all factors considered: condition*time*axis*group (*F* = 4.727, *p* = 0.036). Figure 3 graphically shows the latter result with post-hoc analyses; the threshold of significance was corrected at 0.006 (according to Bonferroni). For the *i*-WG but not the CG significant reductions of upper body acceleration were found along the latero-lateral axis during overground walking outside the parallel bars and along the antero-posterior and latero-lateral axes for walking inside the parallel bars (see Fig. 3).

### Record of falls

During the 6 months after discharge, 4 patients in the i-WG and 9 in the CG reported at least one fall, resulting in an odds ratio of OR = 0.36 (IC95 % = 0.09–1.44). This means that the risk of falling was more than halved in the *i*-WG with respect to the CG. However, this difference was not significantly different between the two groups (*p* = 0.143). An association was also found between subjects who experienced a fall during the six months between T1 and T2 and trunk acceleration assessed at T1: higher normalized trunk accelerations during overground walking at discharge were found in patients who fell after discharge (repeated measures ANOVA: *F*(1,34) = 5.072, *p* = 0.031). These accelerations were also different between the two groups (*F*(1,34) = 4.392, *p* = 0.044) for body axes (*F*(1,34) = 12.048, *p* = 0.001); furthermore, a slightly significant interaction was found between group and fallers/non-fallers (*F*(1,34) = 4.208; *p* = 0.048), as these accelerations were higher in the *i*-WG fallers.

## Discussion

In this study, we aimed to evaluate the effects of walking training performed with the *i*-Walker robotic device on walking performances of patients in the subacute phase following mild/moderate stroke. We found that the use of the *i*-Walker resulted in increased walking speed and walking capacity. This speed increment exceeded the MCID in about half of the patients enrolled in the *i*-WG, a significantly higher number than those in the CG who exceeded this threshold.

Furthermore, after practice the patients trained with the *i*-Walker had increased trunk stability associated with reduced upright gait instabilities, in turn associated with their real number of falls in the community. The increment in speed for both short and long distances was probably achieved because the *i-*Walker allows subjects to walk in a more ecological way than walking training performed between parallel bars, and in a more symmetrical manner than walking using a quadripode.

One mechanism that might underlie the observed improvement of balance competencies (assessed using Tinetti scores and trunk accelerations) might be plasticity dependent recovery [43], which was boosted by intensive task-oriented walking training performed overground and with correct sensory-motor feedback.

The *i*-Walker allows individuals to interact in an ecological environment in a clinical setting and potentially at home and in the community. This is different from therapy using robotics or parallel bars where people are forced to use them in a clinical setting. Involvement of the cognitive system allows integrating a top-down approach with the more conventional bottom-up one during walking, in line with recent findings on walking recovery [44].

Thus, the *i*-Walker could be an effective option for patients who are unable to engage in a training protocol performed on the floor without the continuous help of a physiotherapist for support and balance. At the same time, a body-weight-supported system coupled with an end-effector/ exoskeleton system or treadmill can be helpful for most severely affected patients but is too constrictive for less affected patients because it alters sensory-motor feedback/feedforward signals and thus balance plasticity dependent recovery. This hypothesis is reinforced by the finding that the afferent inputs, which are crucial for postural stability in patients with stroke, are altered in walking-like training such as footplate/treadmill training [45].

From another point of view, conventional walking training with parallel bars or a cane might reinforce asymmetrical posture in a crucial phase of recovery. In fact, it has recently been suggested that spatial asymmetries and motor asymmetries should be treated as the same phenomena by avoiding gestures that reinforce asymmetry [46].

Patients with hemiplegia due to stroke are more prone to falling because of sensory and motor deficit problems and a lack of rapid posture adjustment, which is essential for dynamically stabilized standing. Gait stability is a fundamental parameter that should be measured and trained in patients with stroke, because it is more linked to falls than other walking parameters such as walking velocity and walking inter-limb coordination [47]. Hence, another important finding of our study is the reduction of trunk accelerations related to instabilities in subjects performing *i*-Walker-assisted overground training. Although the number of fallers in the *i*-Walker group was half that in the control group, the difference between the two groups was not statistically different. This was probably because of the few falls that actually occurred, which was to be expected in a sample of 42 subjects. However, at the end of the *i*-Walker training several indicators of upright gait instability (parameters found to be significantly lower in fallers) were significantly reduced. In fact, this was to be expected because dynamic balance deficits are linked to a high risk of falling [48] and alteration of latero-lateral weight shifting is one of the most common causes of falls [49]. Subjects who were trained with *i*-Walker were found to have fewer balance difficulties, especially in the latero-lateral axis, during overground walking (as shown by the statistically significant interaction among condition*time*axis*group). As to antero-posterior instabilities, they were reduced in *i*-WG but only during walking within bars.

This study makes an important contribution to the field of neuromotor recovery and the findings reported here can be translated into rehabilitation practice because of the conjunction between a well-known device, i.e., the *i*-Walker and the advanced technology that allowed us to use it with positive results in mild/moderate haemiparetic subjects.

It should be noted that only patients in the *i*-Walker group showed a decrease in ankle spasticity. It is well known that exercise that promotes orthostatic balance and amount of mobilization has a positive effect on reducing muscle tone after central nervous system damage; by contrast, immobilization or little mobilization leads to an increase in spasticity and contractures [50].

Before concluding, we must mention some limitations of our study. The two main limitations were that the study was registered only after the end of data collection and that the follow-up assessment was limited to records concerning falls and no clinical or instrumental tool was used to assess balance and walking capabilities. Further, the number of falls was self-reported by patients; therefore, it is conceivable that subjects under-reported the incidence of falls. Another limitation of our study is that it is unclear whether the improvements obtained using the *i*-Walker could also have been achieved by the control group if their training had been performed in more variable contexts. In any case, this would have been very difficult to obtain because it would have involved greater effort on the part of the physiotherapist (or the intervention of more than one therapist) and could have led to safety problems related to patients’ falling (or fear of falling). Furthermore, future research should evaluate the effect of the *i*-Walker in a larger sample and should include a follow-up group. Finally, it would be useful to explore the usefulness of the *i*-Walker as an assistive device for use in the home.

## Conclusions

In conclusion, in this study the *i-*Walker was found to be more effective than conventional therapy in improving walking abilities and upright gait stability in patients with mild/moderate deficits due to subacute stroke.
